# Whole-Body Vibration Exercise for Knee Osteoarthritis: A Systematic Review and Meta-Analysis

**DOI:** 10.1155/2015/758147

**Published:** 2015-08-05

**Authors:** Xin Li, Xue-Qiang Wang, Bing-Lin Chen, Ling-Yan Huang, Yu Liu

**Affiliations:** ^1^Key Laboratory of Exercise and Health Sciences of the Ministry of Education, Shanghai University of Sport, Heng Ren Road, No. 200, Yang Pu District, Shanghai 200438, China; ^2^School of Kinesiology, Shanghai University of Sport, Qing Yuan Huan Road, No. 650, Yang Pu District, Shanghai 200438, China; ^3^Department of Sport Rehabilitation, Shanghai University of Sport, Chang Hai Road, No. 399, Yang Pu District, Shanghai 200438, China

## Abstract

*Objectives*. To assess the effects of WBV exercise on patients with KOA.* Methods*. Eight databases including Pubmed, EMBASE, Cochrane Library, CINAHL, Web of Science, the Physiotherapy Evidence Database, CNKI, and Wanfang were searched up to November 2014. Randomized controlled trials (RCTs) of WBV for KOA were eligible. The outcomes were pain intensity, functional performances, self-reported status, adverse events, and muscle strength. A meta-analysis was conducted.* Results*. Five trials with 168 participants provided data for the meta-analysis. No significant difference was shown in pain intensity and self-reported status between WBV and other forms of exercise. Improvement in functional performance (evaluated by BBS; WMD, 2.96; 95% CI, 1.29 to 4.62; *P* = 0.0005) was greater in WBV group, but the other parameters of functional performance (including 6MWT and TGUG) revealed no statistically significant difference. Adverse events were only reported in one trial and no significant difference was discovered in muscle strength. The overall quality of evidence was very low.* Conclusion.* Currently there is only limited evidence that suggested that WBV is effective in the treatment of KOA. Large, well-designed RCTs with better designs are needed.

## 1. Introduction

Osteoarthritis (OA) is a chronic, ingravescent, and degenerative osteoarticular disease with a multifactorial etiology [1]. OA is characterized by arthralgia, stiffness, and limitations in articular function [1–3]. Researchers at the World Health Organization forecast that OA will become the fourth primary cause of disability by the year 2020 [4]. Felson [5] reported that approximately one-third of adults worldwide showed radiological signs of OA. Andrianakos et al. [6] reported significant hand, knee, or hip OA in 8.9% of the adult population. Thus far, the substantial morbidity of knee OA (KOA) has affected approximately 28% of the population aged 45 years and older and 37% of adults older than 65 years of age in the United States [7]. KOA is now one of the leading joint diseases that causes pain, stiffness, loss of physical function, and other adverse effects among adults [8]. The burdens on health care resources and on the economy caused by KOA are substantial [9, 10]. Various forms of exercise, including walking, balance training, resistance training, hydrotherapy, and Tai Chi training, have been investigated as potential methods of KOA management [11].

Whole-body vibration (WBV) exercise is a feasible and curative strength-exercise technique that has received considerable attention in recent years [12]. The vibrations are generated by a vibrating plate and are transmitted from surfaces in contact with the human body to stimulate muscles and tendons. WBV provides a time benefit compared with other traditional resistance exercise programs [13].

During the past decade, some studies have found that WBV may enhance muscle strength and power and may have the potential to be a useful adjuvant in physiotherapy and health care [13]. A large number of studies have assessed the efficiency of WBV on muscle strength and power enhancement [14–20]. Studies have reported that WBV is helpful in improving neuromuscular performance and in providing a safe exercise program for elderly people [19, 21, 22]. WBV has also been found to enhance the multijoint strength performance of the lower limbs during a countermovement jump [23]. A recent meta-analysis also reported that WBV may be useful in increasing the relative basic mobility and balance ability of older adults, particularly among the frail ones [24]. The UK National Institute for Health and Clinic Excellence recommended physical activity and exercise as the core treatment for KOA in enhancing muscle strength and lower extremity multijoint strength [25]. Furthermore, basic balance ability and mobility are highly correlated with the improvement of knee-specific function [26]. Thus, WBV may be beneficial for patients with knee OA.

Ronikeile and Costa [27] reported the benefits of WBV for people with OA by systematically reviewing several studies. Several trials have reported that WBV can alleviate pain, improve balance control, and improve gait pattern and other parameters [28, 29]. However, some trials failed to find significant improvements in pain intensity or other parameters among patients with KOA [30, 31]. Thus, the benefits of WBV on KOA management remain unclear. Thus far, no meta-analysis has evaluated evidence on pain intensity, functional performance, self-reported status, and adverse events. This study aims to evaluate the effect of WBV exercise by comparing WBV with other exercises via meta-analyses for patients with KOA.

## 2. Methods

### 2.1. Literature Search

We authenticated relevant articles by conducting electronic searches on the following databases from the earliest available date to November 2014: PubMed, EMBASE, Cochrane Library, Cumulative Index Nursing and Allied Health Literature, Web of Science, the Physiotherapy Evidence Database, China National Knowledge Infrastructure, and the Wanfang Database. We used terms such as “knee osteoarthritis,” “whole-body vibration,” and “random” in the databases above (File S1 shows the concrete details of the search in Supplementary Material available online at http://dx.doi.org/10.1155/2015/758147). No year, language, or status restrictions were applied to enhance the probability of obtaining interrelated publications that are related to the effect of WBV and KOA.

To identify gray literature, domain experts were consulted and we investigated data that were not represented in the aforementioned databases. Furthermore, the International Controlled Trials Registry Platform was retrieved to search for relevant conference and other literature that might have contained additional data by using “whole-body vibration” and “knee osteoarthritis” as keywords. However, we did not obtain any additional published papers. We also manually screened the reference lists of consilient publications to obtain articles.

### 2.2. Inclusion Criteria

#### 2.2.1. Types of Studies

Randomized controlled trials (RCTs) that compared exercises with or without WBV or compared only WBV exercise with no intervention or placebo group were acceptable. There were no restrictions on year, language, status, or publication date.

#### 2.2.2. Types of Participants

The subjects in the trials included patients who met the diagnostic criteria of definite KOA for at least three months. Subjects exposed to analogous treatments before the study were excluded unless an adequate washout period was described.

#### 2.2.3. Types of Interventions

Trials that compared WBV treatment with no exercise, sham-WBV treatment, or other exercise interventions for KOA were included. Studies in which combinations of interventions were performed were acceptable as long as these combinations were controlled for in the structure of the trial.

#### 2.2.4. Types of Outcome Measures

The results were summarized and analyzed according to four primary outcome classifications: (1) pain intensity, (2) functional performance, for example, Berg balance scale (BBS), 6 min walk test (6MWT), and timed get up and go test (TGUG), (3) self-reported status (using WOMAC scale), and (4) adverse events. The secondary outcome was muscle strength (e.g., extensor peak isokinetic torque, extensor peak isometric torque, and flexor peak isokinetic torque).

### 2.3. Selection of Studies

Two reviewers (Li, X. and Liu, Y.) independently sorted through the articles for potentially relevant titles. Abstracts of all identified records were each screened by two reviewers, and articles were retrieved in full whenever necessary. Any discord was settled by discussing or consulting with another reviewer (Wang, X. Q.) if necessary.

### 2.4. Quality Assessment

To identify the methodological quality of all included trials, the risk of bias tool of the Cochrane Collaboration was applied by two independent authors (Li, X. and Liu, Y.). The evaluated issues included random sequence generation, allocation concealment, blinding of participants and personnel, blinding of outcome assessments, incomplete outcome data, and selective reporting [32]. As far as each domain was concerned, the methods described in each study were examined and potential bias was evaluated in accordance with three grades: low risk, high risk, and unclear risk [27, 33]. Disagreements were resolved after consulting a third independent author (Wang, X. Q.).

### 2.5. Data Extraction

Two authors (Li, X. and Liu, Y.) independently extracted and crosschecked the data acquired from every included trial. The study design (random sequence generation, allocation concealment, blinding of participants and personnel, blinding of outcome assessments, incomplete outcome data, and selective reporting) was then recorded. Any discord was settled by conference to reach unanimity. Authors were contacted directly to acquire the original studies and data when necessary.

### 2.6. Statistical Analysis

The effects of WBV on outcomes of interest were analyzed. Meta-analysis was performed if two or more studies evaluated the same outcome. For each outcome of interest in the selected study, the weighted mean difference (WMD) and 95% confidence interval (CI) were computed. The data was analyzed by Review Manager statistical software (RevMan 5.2, The Cochrane Centre, The Cochrane Collaboration, Denmark) by using a random-effects model. The proportion of variance in the pooled estimates caused by heterogeneity was evaluated by the following parameters: *I* ^2^ index: <25%, low heterogeneity; <75%, moderate heterogeneity; and ≥75%, high heterogeneity [34]. No funnel plots analysis would be performed, if the number of trials pooled in the comparison included in this literature was quite small (maximum of three trials).

The control conditions included both no treatment and sham-WBV treatment and other forms of exercise, including home-based exercise, squat exercise, and balance board exercise. If one trial contained two or more matched groups that performed various exercise programs (e.g., other forms of exercises including home-based exercise, squat exercise, and balance board exercise), the data from the control groups were combined by using the equation in Table 1.

Analyses were possible only for pain intensity, functional performance, self-reported status, and muscle strength because all data on adverse events originated from one article only. All the variables were continuous in the analysis. Therefore, a random-effects model was applied. Two-sided statistical tests were used. *P* < 0.05 or 95% CI was deemed a statistically significant difference [35].

### 2.7. Data Synthesis

Grading of Recommendations Assessment, Development and Evaluation (GRADE) system was used to rate the quality of evidence for each outcome across studies. The system allows for classifying the quality of evidence into high quality, moderate quality, low quality, or very low quality, depending on study design, study limitations (risk of bias), inconsistency, indirectness of study results, imprecision, and publication bias [36]. The judging criteria are listed in Appendix S1.

## 3. Results

### 3.1. Study Selection

**Figure 1:**
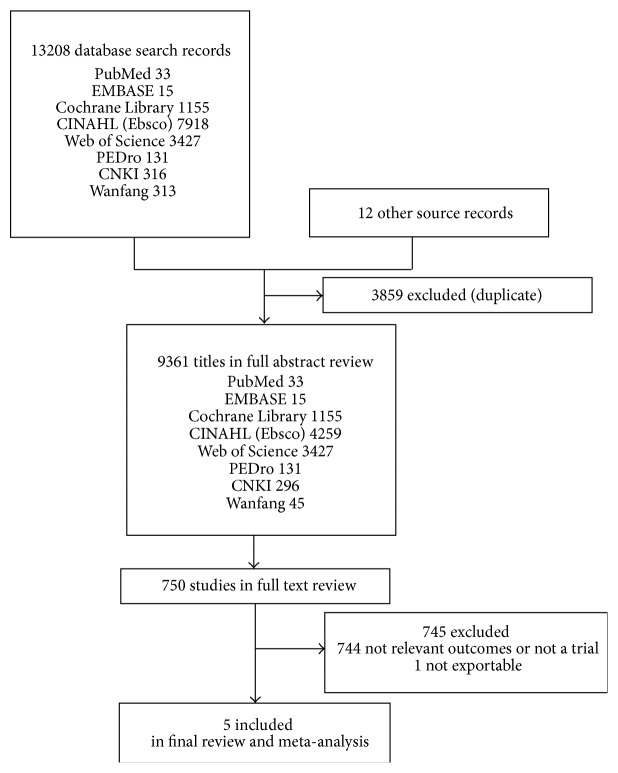
Review flow diagram.

Our first search initially yielded 13220 records. After reviewing the information in the titles and abstracts, 750 papers were read in detail. Finally, 5 RCTs [28–31, 37] (168 participants) with valid outcome data met the inclusion criteria and were included in this analysis. The process of identifying these studies from initial publication searches to final inclusion is illustrated in Figure 1.

### 3.2. Description of Included Studies

Five RCTs with the purpose of examining the efficacy of WBV on KOA [28–31, 37] were included in this meta-analysis. The trials were published in English from 2009 to 2014. The time of intervention ranged from 8 weeks to 12 weeks (median: 9.6 weeks). Among the trials, two trials were from Brazil [29, 31], whereas the three other studies were from Korea [30], Denmark [37], and Finland [28]. Trials recruited participants with KOA mostly from local hospitals and clinics. Across the included studies, three different devices were used for the whole-body vibration exercise. Three studies used vertical vibration [28, 29, 31], one study used side-alternating vibration [37], and one study used multidirectional vibration [30]. Across all five studies the frequency of the vibration used varied from 12 to 40 Hz. Study by study, the reported frequencies of vibration were 12 to 14 Hz [30], 25 to 30 Hz [37], 35 to 40 Hz [29, 31], and 30 Hz [28]. Four studies documented the amplitude of the vibration, reporting amplitudes of 4 mm [29, 31], 2.5–5 mm [30], and 2.5 mm [28]. One study did not report the amplitude of the vibration [37].  Table 2 elaborates the details of the included studies, such as the subjects' characteristics, sample size, intervention, duration of trial period, outcomes, and time points. All the durations of intervention were less than 6 months, and we did not categorize outcomes into different lengths of intervention period.

#### 3.2.1. Participants

We extracted data from 168 participants (number of subjects in the study was from 21 to 52), among whom 64 were exposed to WBV only and 34 received WBV combined with other therapies (11 combined with home-based exercise and 23 combined with squat exercise). The average age of the participants ranged from 58.7 years to 75 years. Subjects were all from hospitals/clinics and were included according to various inclusion criteria.

#### 3.2.2. WBV

The intervention was foremost WBV based, and two [29, 31] of the five trials involved the squat exercises along with the WBV intervention. Exercise duration ranged from 8 weeks to 12 weeks with a mean length of approximately 9.6 weeks. The intervention was performed two or three times a week. Four trials were conducted in the hospital and one was conducted in the clinic. In three of the included studies [29, 31, 37], the vibration time (20–70 s) increased systematically with the number of repetitions (6–9 repetitions). One study was performed when the participants conducted a series of acceleration exercise programs [28]. In another study, participants spent 20 min per session doing WBV [30].

#### 3.2.3. Control Conditions

Two studies used home-based exercises [28, 30] and one study used two independent control interventions, which included squat exercise only and no treatment [29]. One trial used squat exercises as control [31] and the other one used no exercise [37].

#### 3.2.4. Outcomes

Primary outcome measures were pain intensity [28, 30], self-reported status [29, 31, 37], and functional performance [28, 29, 31] (including BBS [29, 31], 6MWT [29, 31], and TGUG [28, 31]). However, none of the included trials reported adverse events. The secondary outcome measure was muscle strength [28, 30, 31, 37].

### 3.3. Methodological Quality of Included Trials

Following recommendations, the risk of bias was assessed in every included trial and we found that the amount of methodological details varied. For example, several quality indexes were insufficiently described in the articles (Table 3). All of the five trials [28–31, 37] described the randomization method that was being used, but three [28, 30, 31] reported no information on treatment allocation concealment. The blinding of assessors was used in four studies [28, 29, 31, 37]. None of the five included trials [28–31, 37] met the requirements for the blinding of participants. However, in view of the characteristics in therapeutic interventions and direct participant-personnel involvement, the blinding of participants was deemed unfeasible, although the absence of blinding must be viewed as a potential source of bias. By contrast, a low risk of bias about incomplete outcome data was only reported in one trial. It was unclear if all five trials were free of selective outcome reporting. Since the assessment of publication bias was only feasible for those comparisons that included a minimum of three studies, we failed to assess other bias. Overall, the methodological quality of the five included trials was judged to be highly risky. What is more, given that the number of trials pooled in the comparison included in this literature was quite small (maximum of three trials), no funnel plots analysis was performed. The quality of evidence was downgraded by very serious study limitations and imprecision. Therefore, there was very low quality evidence overall.

### 3.4. Effectiveness

#### 3.4.1. Pain Intensity

Two trials randomly assigned patients to undergo WBV combined with/without home-based exercise or home-based exercise only [28, 30]. Of these two studies, one trial evaluated pain intensity by using a 100 mm visual analog scale (VAS) [28] and the other by using a 0-point to 10-point numerical rating scale (NRS) [30]. Moderate to high correlations exist between these two different measures [38].

**Figure 2:**
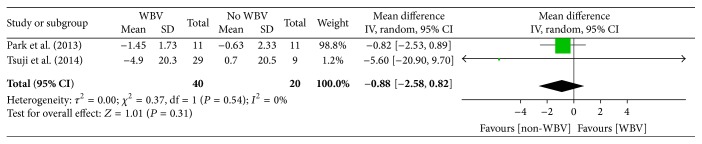
Pain intensity (evaluated by VAS or NRS) for WBV combined with other forms of exercises.

An improvement in pain intensity was observed for both trials but did not reach statistical significance in the meta-analysis (−0.88 points (CI, −2.58 to 0.82), *P* = 0.31) (Figure 2). This result was consistent with the conclusion of Tsuji et al. [28]. The analysis in this study also showed that heterogeneity is low (*I* ^2^ = 0%).

#### 3.4.2. Functional Performance

In the five concluded studies [28–31, 37], functional performance was evaluated by various methods including BBS [29, 39], 6MWT [29, 39], TGUG test [28, 31], 10 m walking time [29], KWOMAC [30], and LSS [30].

**Figure 3:**
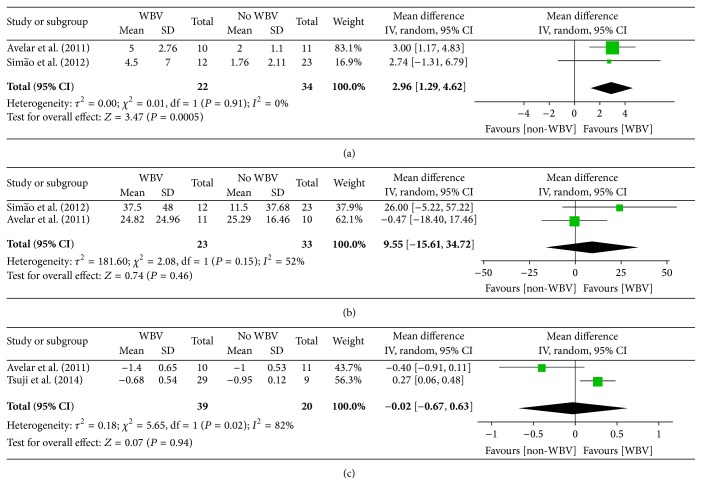
Functional performance (evaluated by BBS, 6MWT, and TGUG) for WBV compared with other forms of exercises.

BBS was significantly improved with WBV exercise compared with other interventions without WBV (2.96 points (CI, 1.29 to 4.62), *P* = 0.0005 < 0.001) (Figure 3(a)). One study [39] comparing WBV combined with squat exercise and squat-only exercise found negative effects for WBV combined with squat exercise on improving BBS and 6MWT. By contrast, another study [29] comparing WBV combined with squat exercise and squat-only exercise, as well as a no exercise group, reported a positive effect. This result is in accordance with the conclusions of one included trial [29].

Although we failed to find a significant difference in 6MWT (9.55 points (CI, −15.61 to 34.72), *P* = 0.46) (Figure 3(b)), this result was in accordance with the conclusions of another trial [31]. By contrast, the heterogeneity of 6MWT was substantially moderate (*I* ^2^ = 52%). This outcome might be caused by differences in the control methods between the two analyzed trials [29, 31]. The squat-only exercise routine was used as the control treatment in one trial [31], whereas the other trial [29] had other different no-treatment groups.

Two of the five included studies [28, 31] reported the effect of WBV according to functional ability by TGUG. One study [31] reported a negative result in TGUG, whereas another one [28] reported that WBV had a positively significant difference in decreasing TGUG time. On the basis of these two trials, we failed to record a statistically significant difference in the meta-analysis between WBV and other exercise or placebo exercise routines in decreasing TGUG time (−0.02 points (CI, −0.67 to 0.63), *P* = 0.94) (Figure 3(c)).

The statistical heterogeneity for TGUG was relatively high (*I* ^2^ = 82%). This result might be caused by the different control methods used in these two studies. Tsuji et al. [28] compared WBV with exercise at home, whereas another study [31] chose the squatting-only group as the control group in contrast to the combination of WBV and squat exercise.

#### 3.4.3. Self-Reported Status

Among the included trials, three studies [29, 31, 37] (108 patients) demonstrated WBV and other forms of exercise by randomly assigning patients (58 and 50 patients, resp.); however, we failed to obtain the original data from one trial [37]. Therefore, the results considered self-reported status as determined by WOMAC.

**Figure 4:**
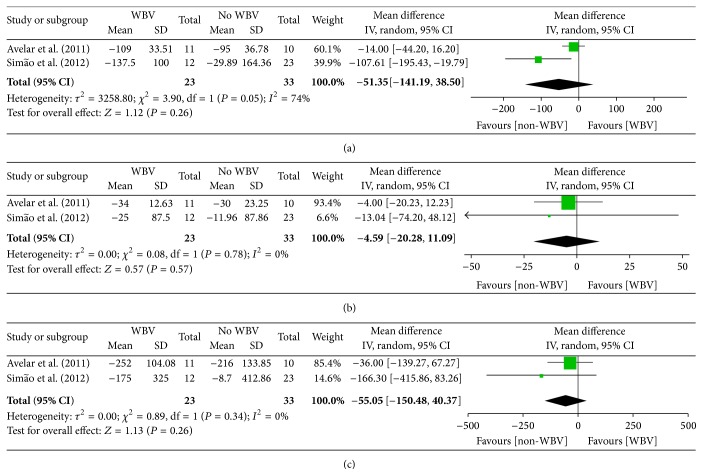
Self-reported status (evaluated by WOMAC-pain, WOMAC-stiffness, and WOMAC-function) for WBV compared with other forms of exercises.

A meta-analysis of three trials [29, 31, 37] showed that WBV and other forms of exercise have no significant difference in terms of WOMAC-pain (−51.35 points (CI, −141.19 to 38.50), *P* = 0.26) (Figure 4(a)), WOMAC-stiffness (−4.59 points (CI, −20.28 to 11.09), *P* = 0.57) (Figure 4(b)), and WOMAC-function (−55.05 points (CI, −150.48 to 40.37), *P* = 0.26) (Figure 4(c)).

Substantially high levels of heterogeneity (*I* ^2^ = 74%) in WOMAC-pain were shown in the pooled studies [29, 31, 37] probably because of the differences among the two included studies with respect to the participant populations. The baseline level of pain, stiffness, and function observed by Simão et al. [29] was lower than those demonstrated by Avelar et al. [31].

#### 3.4.4. Muscle Strength

**Figure 5:**
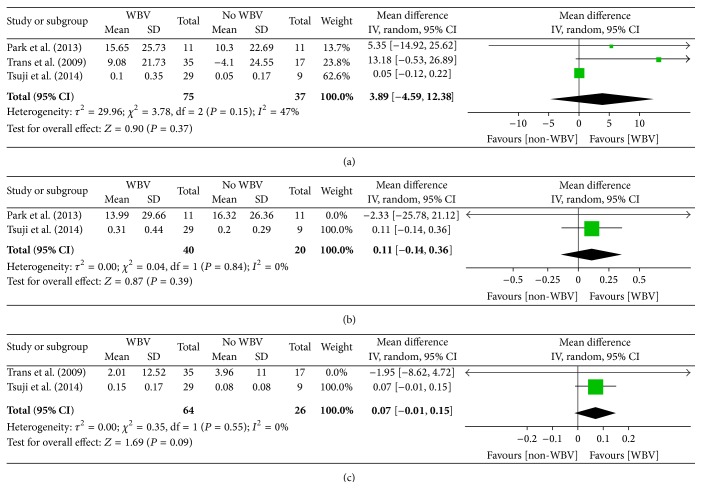
Muscle strength (evaluated by extensor peak isokinetic torque, extensor peak isometric torque, and flexion peak isokinetic torque) for WBV compared with other forms of exercises.

Three RCTs [28, 30, 37] with 112 participants receiving WBV with home-based exercise and another form of exercise reported data about muscle strength, including extensor peak isokinetic torque [28, 30, 37], extensor peak isometric torque [28, 30], and flexor peak isokinetic torque [28, 37]. The meta-analyses of these trials failed to reveal significant differences in extensor peak isokinetic torque (3.89 points (CI, −4.59 to 12.38)) (Figure 5(a)), extensor peak isometric torque (0.11 points (CI, −0.14 to 0.36)) (Figure 5(b)), or flexor peak isokinetic torque (0.07 points (CI, −0.01 to 0.15)) (Figure 5(c)).

However, significant heterogeneity for extensor peak isokinetic torque was observed when WBV was compared with other types of exercises. The heterogeneity of the extensor peak isokinetic torque was moderate (*I* ^2^ = 47%). Visual inspection of the forest plot revealed that three trials [28, 30, 37] in which the control intervention was primarily performed by participants at home were the greatest possible source of heterogeneity.

#### 3.4.5. Adverse Events

Only one RCT [39] with 28 participants receiving WBV or squat exercise reported no serious adverse effects, such as severe injuries or adverse cardiovascular effects. The evidence on adverse events of WBV is quite limited.

## 4. Discussion

Ronikeile and Costa [27] conducted a systematic review of WBV and its benefits for subjects with OA. They found that there were few publications regarding both WBV and OA, and the reported study protocols were diverse. As far as we know, this meta-analysis is the first one to evaluate the performance of WBV in KOA management.

When we applied meta-analysis to assess the consistency of evidence for the efficiency of WBV over other interventions in patients with KOA [40–42], the results were as follows:

1. The meta-analysis showed no statistically significant differences in pain intensity evaluated by NRS and VAS [28, 30] between WBV combined with squat exercise and squat exercise alone.
2. We also found no significant differences in functional performance and self-reported status. However, a statistically significant difference was found between WBV combined with squat exercise and squat exercise alone for BBS on the basis of a comparison between two trials with less than 60 patients [29, 31]. The two trials both had high risks for incomplete outcomes, and allocation concealment was only performed in one of these two trials [29].
3. WBV is as effective as other forms of exercises in enhancing muscle strength according to an evaluation of 133 patients in four trials that had opposite results and significant differences with regard to sample size [28, 30, 31, 37].
4. However, the absence of significant differences between WBV and other forms of exercise might be caused by the poor quality of studies providing data for some of the comparisons in the analysis.

### 4.1. Strengths

Our study has several strengths. We collected the data by applying a large-scale search scheme on the basis of common routines: retrieval of diversiform citation databases, recruitment of professors for published or unpublished studies, searching for document registries, and abstracting the references of the included trials. Furthermore, we did not set any limitations on year, language, status, and publication date. This strategy meant that our summary should represent the present status of all relevant research. Our review was also applied on the basis of previously published protocols and methodological schemes. The consequences of the primary outcomes of our review, including pain intensity, functional performance, and self-reported status, were reasonable. Two of the studies included in this analysis [28, 30] had an evaluation of pain intensity, four of the studies included in this literature [28, 29, 31, 37] had a measure of functional performance, and three trials [29, 31, 37] reported self-reported status although only two of them provided original data for the meta-analysis of self-reported status [29, 31].

#### 4.1.1. Study Limitations

First, because of the methodological weaknesses in all of the studies [28–31, 37] included in our analysis, this systematic review is fundamentally limited. The risk of bias was considered high in the included studies. The most common methodological drawback in the included trials was the lack of patient and personnel blinding. However, given the characteristics of the therapeutic interventions, keeping the patients blind to the study procedures was almost impossible.

Second, we were unable to retrieve all of the original data that was needed for the analysis of the included studies [37]. The lack of some of the original data must be considered a limitation of this review because whether or not these missing results could alter the conclusion remains unknown.

Third, the population of the included trials was relatively small to the extent that none of the studies in this review enrolled 100 or more participants. These trials [28–31, 37] with a small population size were insufficient to show a distinction, which was unlikely to be found between the effectiveness of WBV and other intervention methods. In any case, the inclusion of only small trials affects not only the findings but also the statistical strength of those findings of our study, for instance, our failure to observe statistically significant differences regarding pain intensity, self-reported status, and muscle strength.

Last, the number of RCTs published in this field of study is limited. This limitation might increase the possibility of publication bias. The five trials included were significantly low in therapeutic management research, and the outcomes were notably varied between any two trials. Even though we conducted further searches for unpublished studies, no viable studies were obtained.

### 4.2. Implications for Research

We cannot resolve all the issues involved in understanding the effects of WBV in KOA in one paper. However, large-scale studies with improved designs should be performed to confirm the effectiveness of WBV for KOA because of the limitations in the current trial literature [28–31, 37]. First, a disciplined approach to randomization should be adopted, and the intervention allocation should be adequately concealed. Second, further studies should have well-designed methodologies, particularly with regard to the duration of WBV: none of the included studies lasted for more than six months. Moreover, studies should investigate possibility that the length of intervention duration may affect the relative performance of WBV compared to other interventions in managing KOA. Fourth, outcome measures for further trials might usefully focus on pain intensity, functional performance, self-reported status, and adverse events. Furthermore, long-term follow-up would be useful to estimate whether any improvements owing to WBV are persistent, and if so, for how long.

## 5. Conclusions

No differences were found in decreasing pain intensity or improving self-reported status, in addition to muscle strength enhancement compared with other forms of exercise. However, WBV combined with squat exercise was more efficacious than squat exercise alone in increasing the level of functional BBS performance. Nevertheless, interpretation of our results has to be cautious because of the limitations of the included trials, such as methodological drawbacks and the small population size.

## Supplementary Material

Appendix S1: The judging criteria of Grading of Recommendations Assessment, Development and Evaluation (GRADE) system.
Supplementary fileS1: Search strategies for all databases.
ChecklistS1 PRISMA 2009: Checklist for this meta-analysis.

## Figures and Tables

**Table 1:** 

	Group 1	Group 2	Combined groups
Sample size	*N*_1_	*N*_2_	*N*_1_ + *N*_2_
Mean	*M*_1_	*M*_2_	(*N*_1_ *M*_1_ + *N*_2_ *M*_2_)/(*N*_1_ + *N*_2_)
SD	SD_1_	SD_2_	$\sqrt{\left(\left(N_{1}-1\right){SD}_{1}^{2}+\left(N_{2}-1\right){SD}_{2}^{2}+(N_{1}N_{2}/{(N}_{1}+N_{2}))\left(M_{1}^{2}+M_{2}^{2}-2M_{1}M_{2}\right)\right)/\left(N_{1}+N_{2}-1\right)}$

**Table 2:** Characteristics of trials included in systematic review.

Article (year)	Patients characteristic, sample size	Intervention	Duration of trial period	Outcomes	Time point
Avelar et al. (2011) [31]	Source: 21 elderly volunteers (G1 = 11, G2 = 10) Mean age (SD): G1 = 75 y (5), G2 = 71 y (4)	G1: WBV + SE G2: SE only	3 Ts a week for 12 Ws	Functional performance (balance: BBS; functional mobility: TGUG; muscle conditions: CST; walking performance: 6 MWT) Self-reported status of KOA (WOMAC)	12 Ws

Park et al. (2013) [30]	Source: 22 female ambulatory community-based patients (G1 = 11, G2 = 11) Mean age (SD): G1 = 62.5 y (5.66), G2 = 60.0 y (6.22)	G1: WBV + HBE G2: HBE only	3 Ts a week for 8 Ws	Pain intensity (NRS) Functional scales (KWOMAC and LSS) Muscle strength (ISK, ISM) (Both sides) Dynamic balance (SBCS)	8 Ws

Simão et al. (2012) [29]	Source: 35 elderly subjects with KOA (G1 = 12, G2 = 11, and G3 = 12) Mean age (SD): G1 = 75 y (7.4), G2 = 69 y (3.7), and G3 = 71 y (5.3)	G1: WBV + SE G2: SE only G3: no exercise	3 Ts a week for 12 Ws	Self-reported physical function (WOMAC) Functional performance tests (BBS, GST, and 6MWT) Plasma soluble tumor necrosis factor- receptors: 1 (sTNFR1) and 2 (sTNFR2)	12 Ws

Trans et al. (2009) [37]	Source: 52 female patients diagnosed with KOA in an outpatient clinic (G1 = 17, G2 = 18, and G3 = 17) Mean age (SD): G1 = 61.5 y (9.2), G2 = 58.7 y (11.0), and G3 = 61.1 y (8.5)	G1: WBV on a stable platform G2: WBV on a balance board G3: control group	2 Ts a week for 8 Ws	Muscle strength (extension/flexion ISK, ISM) Proprioception (TDPM) Self-reported disease status (WOMAC)	8 Ws

Tsuji et al. (2014) [28]	Source: 38 female patients diagnosed with KOA were recruited via advertisements (G1 = 29, G = 9) Mean age (SD): G1 = 62.1 y (5.5), G2 = 60.9 y (4.6)	G1: WBV G2: HBE	3 Ts a week for 8 Ws	Knee strength and power (ISM, ISK/isokinetic flexion torque of knee, isokinetic extension average power, and isokinetic flexion average power) Self-reported knee function: JKOM degree of pain (VAS) Pain and stiffness in knees (JKOM), condition in daily life (JKOM), general activities (JKOM), and health condition (JKOM) Radiological severity of OA: KL scale	8 Ws

KOA, knee osteoarthritis; WBV, whole-body vibration; SE, squatting exercise; HBE, home-based exercise; BBS, Berg balance scale; TGUG, timed get up and go test; CST, chair stand test; 6MWT, 6 min walk test; WOMAC, The Western Ontario and McMaster Universities Osteoarthritis Index; NRS, numerical rating scale; KWOMAC, Korean Western Ontario McMaster score; LSS, Lysholm scoring scale; ISK, isokinetic torque of knee extensor; ISM, isometric torque of knee extensor; SBCS, standing balance control scores; GST, gait speed test; sTNFR, plasma soluble tumor necrosis factor-*α* receptors; TDPM, threshold for detection of passive movement; JKOM, Japanese Knee Osteoarthritis Measure; KL, Kellgren-Lawrence; VAS, visual analog scale; Ts, times; Ws, weeks.

**Table 3:** The Cochrane Collaboration's tool of assessing risk of bias for methodological assessment.

Article (year)	Random sequence generation	Allocation concealment	Blinding of participants and personnel	Blinding of outcome assessments	Incomplete outcome data	Selective reporting
Avelar et al. (2011) [31]	Low	High	High	Low	High	Unclear
Park et al. (2013) [30]	Low	High	High	Unclear	Low	Unclear
Simão et al. (2012) [29]	Low	Low	High	Low	High	Unclear
Trans et al. (2009) [37]	Low	Low	High	Low	High	Unclear
Tsuji et al. (2014) [28]	Low	High	High	Low	High	Unclear

## Abbreviations

6MWT: 6 min walk test
BBS: Berg balance scale
CI: Confidence interval
CST: Chair stand test
GST: Gait speed test
ISK: Isokinetic torque of knee extensor
ISM: Isometric torque of knee extensor
JKOM: Japanese Knee Osteoarthritis Measure
KL: Kellgren-Lawrence
KOA: Knee osteoarthritis
KWOMAC: Korean Western Ontario McMaster score
LSS: Lysholm scoring scale
NRS: Numerical rating scale
OA: Osteoarthritis
RCTs: Randomized controlled trials
SBCS: Standing balance control scores
SD: Standard deviation
SE: Squatting exercise
sTNFR: Plasma soluble tumor necrosis factor-*α* receptors
TDPM: Threshold for detection of passive movement
TUGT: Timed get up and go test
VAS: Visual analog scale
WBV: Whole-body vibration
WOMAC: The Western Ontario and McMaster Universities Osteoarthritis Index.
