# Erratum to “Whole-Body Vibration Exercise for Knee Osteoarthritis: A Systematic Review and Meta-Analysis”

**DOI:** 10.1155/2015/636435

**Published:** 2015-11-09

**Authors:** Xin Li, Xue-Qiang Wang, Bing-Lin Chen, Ling-Yan Huang, Yu Liu

**Affiliations:** ^1^Key Laboratory of Exercise and Health Sciences of the Ministry of Education, Shanghai University of Sport, Heng Ren Road, No. 200, Yang Pu District, Shanghai 200438, China; ^2^School of Kinesiology, Shanghai University of Sport, Qing Yuan Huan Road, No. 650, Yang Pu District, Shanghai 200438, China; ^3^Department of Sport Rehabilitation, Shanghai University of Sport, Chang Hai Road, No. 399, Yang Pu District, Shanghai 200438, China

In our paper entitled “Whole-Body Vibration Exercise for Knee Osteoarthritis: A Systematic Review and Meta-Analysis” [1], there was an error in the journal name and the authors' names of reference number [27] mentioned in the references list. We have now provided new corrected reference number [27]:

 [27] P. R. da Costa, D. da Cunha Sá-Caputo, A. Arnóbio et al., “Whole-body vibration and benefits for people with osteoarthritis: a system review,”* International Journal of Medicine and Medical Sciences*, vol. 6, no. 9, pp. 201–210, 2014.

These corrections do not change any conclusion or findings of the paper.

## References

[1] Li X., Wang X.-Q., Chen B.-L., Huang L.-Y., Liu Y. (2015). Whole-body vibration exercise for knee osteoarthritis: a systematic review and meta-analysis. *Evidence-Based Complementary and Alternative Medicine*.

