# Supplementary material for: Whole-Body Vibration Exercise for Knee Osteoarthritis: A Systematic Review and Meta-Analysis
Source: Evid Based Complement Alternat Med. 2015 Aug 5;2015:758147. doi: 10.1155/2015/758147 (PMC4540999; doi:10.1155/2015/758147)
Supplement: Supplementary file 1 — Appendix S1: The judging criteria of Grading of Recommendations Assessment, Development and Evaluation (GRADE) system. Supplementary fileS1: Search strategies for all databases. ChecklistS1 PRISMA 2009: Checklist for this meta-analysis. [file 758147.f1.zip › (Appendix S1) The judging criteria of the GRADE.doc]

**Appendix S1.** **The judging criteria of the GRADE (Grading of Recommendations Assessment, Development and Evaluation) system**

***Study limitations.*** Study limitations were judged based on the results of assessing risk of bias. As subjective outcome is influenced by lack of double blinding and inadequate concealment [43], studies involving one of these risks were considered to have moderate risk of bias and those involving both risks were considered to have high risk of bias. A cut-off value of 25% of total participants was used to judge if a body of evidence would be downgraded [44]. When >25% of the participants was from studies with high risk of bias, two levels were downgraded (very serious limitations). When >25% of participants was from studies with moderate and high risk of bias and those from high risk of bias were 0-25%, one level was downgraded (serious limitations).

***Inconsistency.*** Inconsistency was judged by the degree of heterogeneity (*I^2^* > 40%). If there was evidence of heterogeneity, one level was downgraded.

***Indirectness.*** Indirectness which refers to the generalizability of findings was decided by the characteristics of participants. If there was clinical heterogeneity in participants (e.g. 50% of adults and 50% of children), one level was downgraded.

***Imprecision.*** Imprecision was decided by the number of subjects (<200 participants) [45]. If there were less than 200 participants in total, one level was downgraded.

***Publication bias.*** Publication bias was judged by funnel plot and sources of funding. If the funnel plot demonstrated asymmetry in visual inspection and most of the studies (>75%) were industry sponsored, likely to be industry sponsored or the authors shared another conflict of interest, one level was downgraded. It was considered only when there was obvious evidence.
