# Supplementary material for: Whole-Body Vibration Exercise for Knee Osteoarthritis: A Systematic Review and Meta-Analysis
Source: Evid Based Complement Alternat Med. 2015 Aug 5;2015:758147. doi: 10.1155/2015/758147 (PMC4540999; doi:10.1155/2015/758147)
Supplement: Supplementary file 1 — Appendix S1: The judging criteria of Grading of Recommendations Assessment, Development and Evaluation (GRADE) system. Supplementary fileS1: Search strategies for all databases. ChecklistS1 PRISMA 2009: Checklist for this meta-analysis. [file 758147.f1.zip › (Supplementary file S1) Search strategy.doc]

Whole-body vibration exercise for osteoarthritis of the knee: a systematic review and meta-analysis

**Search strategies for all databases**

1. Search Strategy for PubMed:

#1 "Single-Blind Method"[Mesh] OR "Double-Blind Method"[Mesh] OR "Randomized Controlled Trials as Topic"[Mesh] OR "Randomized Controlled Trial" [Publication Type] OR "Intention to Treat Analysis"[Mesh] OR "Controlled Clinical Trials as Topic"[Mesh] OR "Clinical Trials as Topic"[Mesh] OR "Clinical Trial" [Publication Type]

#2 "random*"[Text Word] OR allocation[Text Word] OR "random allocation"[Text Word] OR placebo[Text Word] OR single blind[Text Word] OR double blind[Text Word] OR "randomized controlled trial*"[Text Word] OR RCT[Text Word]

#3 randomized controlled trial[Publication Type]

#4 #1 OR #2 OR #3

#5 animals NOT humans

#6 #4 NOT #5

#7 osteoarthritis [Mesh] OR arthritis [Mesh] OR arthralgia [Mesh] OR joint disease [Mesh] OR osteoarthritis [Mesh] OR osteoarthrosis [Mesh] OR gonarthrosis [Mesh] OR degenerative arthritis [Title/Abstract] OR arthritis [Title/Abstract] OR degenerative[Title/Abstract] OR degenerative joints[Title/Abstract]

#8 "whole body vibration"[All Fields] OR "whole body"[All Fields] AND "vibration"[Mesh] OR "vibration"[All Fields]

#9 #6 AND #7 AND #8

2. Search Strategy for EMBASE:

#1 'randomization'/exp OR 'placebo'/exp OR 'placebo effect'/exp OR 'single blind procedure'/exp OR 'double blind procedure'/exp OR 'randomized controlled trial'/exp OR 'randomized controlled trial (topic)'/exp OR 'controlled clinical trial'/exp OR 'controlled clinical trial (topic)'/exp OR 'clinical trial'/exp OR 'clinical trial (topic)'/exp

#2 random*:ab,ti OR allocation:ab,ti OR "random allocation":ab,ti OR placebo:ab,ti OR single blind:ab,ti OR double blind:ab,ti OR randomised controlled trial*:ab,ti OR randomized controlled trial*:ab,ti OR RCT:ab,ti OR clinical trial*:ab,ti

#3 #1 OR #2

#4 osteoarthritis/exp OR arthritis/exp OR arthralgia/exp OR joint disease/exp OR joint/exp OR disease/exp OR osteoarthritis:ab,ti OR osteoarthrosis:ab,ti OR gonarthrosis:ab,ti OR degenerative arthritis:ab,ti OR arthritis:ab,ti OR degenerative:ab,ti OR degenerative joints:ab,ti (**21,442**)

#5 'whole body vibration'/exp OR 'whole body vibration' OR 'whole body'/exp OR 'whole body' OR 'vibration'/exp OR ' vibration'

#6 #3 AND #4 AND #5

3. Search Strategy for Cochrane Library

#1 "random*" or allocation or "random allocation" or placebo or single blind or double blind or "randomized controlled trial*" or RCT or "clinical trial*"

#2 [publication type]randomized controlled trial or clinical trial

**#3 #1OR#2**

#4 osteoarthritis OR arthritis OR arthralgia OR joint disease OR joint OR disease OR osteoarthrosis OR gonarthrosis OR degenerative arthritis OR degenerative OR degenerative joints:ti,ab,kw

#5 'whole body vibration' OR 'whole body' OR 'vibration'

#6 #3 And #4 And #5

4. Search Strategy for CINAHL (Ebsco)

S1 MH("Random Assignment" OR "Placebos" OR "Placebo Effect" OR "Single-Blind Studies" OR "Double-Blind Studies" OR "Triple-Blind Studies" OR "Randomized Controlled Trials" OR "comparative studies" OR "Evaluation Research" OR "Prospective Studies" OR "crossover Design" OR "Prospective Studies" OR "Clinical Trials" OR "Clinical Trial Registry")

S2 TX(random$ OR allocation OR "random allocation" OR placebo$ OR single blind OR double blind OR "randomi?ed controlled trial*" OR "controlled clinical trial*" OR "comparative study" OR "evaluation stud*" OR "follow-up stud*" OR "prospective stud*" OR "cross-over stud*" OR control$ OR prospectiv$ OR volunteer$ OR "RCT" OR "clinical trial*")

S3 PT( randomized controlled trial OR "clinical trial*")

S4 S1 OR S2 OR S3

S5 AB(osteoarthritis OR arthritis OR arthralgia OR joint disease OR joint OR disease OR osteoarthrosis OR gonarthrosis OR degenerative arthritis OR degenerative OR degenerative joints)

S6 AB(whole body vibration OR whole body OR vibration)

S7 S4 AND S5 AND S6

5. Search Strategy for web of science

#1 TS=("random*" OR allocation OR "random allocation" OR placebo OR single blind OR single blind method OR double blind OR double blind method OR "randomized controlled trial*" OR "randomised controlled trial*" OR "RCT" OR "clinical trial*")

#2 TS=( osteoarthritis OR arthritis OR arthralgia OR joint disease OR joint OR disease OR osteoarthrosis OR gonarthrosis OR degenerative arthritis OR degenerative OR degenerative joints)

#3 =( whole body vibration OR whole body OR vibration)

#4 #1 AND #2 AND #

Timespan=All years. Databases=SCI-EXPANDED, SSCI, A&HCI, CPCI-S, CPCI-SSH.

6. Search Strategy for PEDro

Abstract & Title: whole body vibration

Therapy: no selection

Problem:no selection

Body part: no selection

Subdiscipline: no selection

Topic:no selection

Method: clinical trial

Author/Association:no limited

Title Only:no limited

Source:no limited

Published Since:no limited

New records added since:no limited

Score of at least:no limited

Return:50(records at a time)

When searching: match all search terms (AND)

7. Search Strategy for CNKI

#1 SU=随机 OR SU=随机分配 OR SU=随机对照 OR SU=对照 OR SU=盲法 OR SU=单盲 OR SU=双盲 OR SU=随机对照试验 OR SU=随机对照研究 OR SU=临床试验 OR SU=临床观察 OR SU=临床研究 （精确匹配）

#2 SU=骨关节炎OR SU=关节炎 OR SU=关节痛 OR SU=关节疾病 OR SU=关节 OR SU=关节僵硬OR SU=骨关节病 OR SU=膝关节炎 OR SU=退行性关节炎OR SU=退化的 OR SU=退化性关节炎（精确匹配）

#3 SU=振动 OR SU=全身振动 OR SU=振动训练 OR SU=全身训练

#4 #1 AND #2 AND #3

8.Search Strategy for CBM

#1 "随机分配"[扩展：不加权] OR "对照组"[扩展：不加权] OR "单盲法"[扩展：不加权] OR "双盲法"[扩展：不加权] OR "随机对照试验"[扩展：不加权] OR "临床对照试验"[扩展：不加权] OR "临床试验"[扩展：不加权]

#2 "随机对照试验(主题)"[扩展：不加权] OR "临床对照试验(主题)"[扩展：不加权] OR "临床试验(主题)"[扩展：不加权]

#3 "随机"[全字段：智能] OR "随机分配"[全字段：智能] OR "随机对照"[全字段：智能] OR "对照"[全字段：智能] OR "盲法"[全字段：智能] OR "单盲"[全字段：智能] OR "双盲"[全字段：智能] OR "随机对照试验"[全字段：智能] OR "随机对照试验（文献类型）"[全字段：智能] OR "随机对照研究"[全字段：智能] OR "临床试验"[全字段：智能] OR "临床观察"[全字段：智能] OR "临床研究"[全字段：智能]

#4 (#1) OR (#2) OR (#3)

#5 "膝骨关节炎"[扩展：不加权]

#6 "骨关节炎"[中文标题：智能] OR "关节炎"[中文标题：智能] OR "关节痛"[中文标题：智能] OR "关节疾病"[中文标题：智能] OR "关节"[中文标题：智能] OR "关节僵硬"[中文标题：智能] OR "骨关节病"[中文标题：智能] OR "膝关节炎"[中文标题：智能] OR "退行性关节炎"[中文标题：智能] OR "退化的"[中文标题：智能] OR "退化性关节炎"[中文标题：智能]

#7 "骨关节炎"[摘要：智能] OR "关节炎"[摘要：智能] OR "关节痛"[摘要：智能] OR "关节疾病"[摘要：智能] OR "关节"[摘要：智能] OR "关节僵硬"[摘要：智能] OR "骨关节病"[摘要：智能] OR "膝关节炎"[摘要：智能] OR "退行性关节炎"[摘要：智能] OR "退化的"[摘要：智能] OR "退化性关节炎"[摘要：智能]

#8 "骨关节炎"[关键词：智能] OR "关节炎"[关键词：智能] OR "关节痛"[关键词：智能] OR "关节疾病"[关键词：智能] OR "关节"[关键词：智能] OR "关节僵硬"[关键词：智能] OR "骨关节病"[关键词：智能] OR "膝关节炎"[关键词：智能] OR "退行性关节炎"[关键词：智能] OR "退化的"[关键词：智能] OR "退化性关节炎"[关键词：智能]

#9 (#5) OR (#6) OR (#7) OR (#8)

#10 "振动"[扩展：不加权]

#11 "振动"[中文标题：智能] OR "全身振动"[中文标题：智能] OR "振动训练"[中文标题：智能] OR "全身训练"[中文标题：智能]

#12 "振动"[摘要：智能] OR "全身振动"[摘要：智能] OR "振动训练"[摘要：智能] OR"全身训练"[摘要：智能]

#13 "振动"[关键词：智能] OR "全身振动"[关键词：智能] OR "振动训练"[关键词：智能] OR "全身训练"[关键词：智能]

#14 (#10) OR (#11) OR (#12) OR (#13)

#15 (#4) AND (#9) AND (#14)

9.**Search Strategy for Wanfang Database**

#1 主题:("随机" + "随机分配" + "随机对照" + "对照" + "盲法" + "单盲" + "双盲" + "随机对照试验" + "临床试验" + "临床观察")

#2 主题:("骨关节炎" + "关节炎" + "关节痛" + "关节疾病" + "关节" + "关节僵硬" + "骨关节病" + "关节疾病" + "膝关节炎" + "退行性关节炎" + "退化的"+ "退化性关节炎")

#3 主题:("振动" + "全身振动" + "振动训练" + "全身振动")

#4 (#1) * (#2) * (#3)
